# Childhood Tumors around the Knee Revisited: Predilection Sites for Most Entities Confirmed

**DOI:** 10.3390/jcm13154405

**Published:** 2024-07-27

**Authors:** Sebastian Breden, Simone Beischl, Florian Hinterwimmer, Sarah Consalvo, Ulrich Lenze, Rüdiger von Eisenhart-Rothe, Florian Pohlig, Carolin Knebel

**Affiliations:** Department of Orthopedics and Sports Orthopedics, Klinikum Rechts der Isar, Technical University of Munich, 81675 Munich, Germany

**Keywords:** pediatric cancer, sarcoma, musculoskeletal tumor, distribution pattern, localization, knee, bone, soft tissue

## Abstract

**Background:** The diagnostic work-up of musculoskeletal tumors is a multifactorial process. During the early phase, differential diagnoses are made using basic radiological imaging. In this phase, part of the decision making is based on the patient’s age, as well as the incidence and predilection sites of different entities. Unfortunately, this information is based on older and fragmented data. In this study, we retrospectively evaluated all soft-tissue and bone tumors around the knee in children treated at our tertiary center in the last 20 years, with the aim of verifying the data used today. **Methods:** In this retrospective study, the databank of our tertiary center was used to give an overview of treated tumors around the knee in children. **Results:** We were able to include 224 children with bone and soft-tissue tumors around the knee. The cohort consisted of 184 bone tumors, of which 144 were benign and 40 malignant. The 40 soft-tissue tumors comprised 30 benign and 10 malignant masses. The most common lesions were osteochondromas (88) in the bone and tenosynovial giant-cell tumors (12) in the soft tissue. **Conclusions:** With this original work, we were able to verify and supplement earlier studies, as well as deepen our insight into these very rare diseases.

## 1. Introduction

Cancer in children, particularly musculoskeletal malignancies, is rare. However, malignant tumors are the second most important cause of death before the age of 18 [1]. Despite higher numbers of malignant primary bone tumors in children compared to adults, usually, less than 1000 new cases are diagnosed in the US per year [2]. Soft-tissue tumors are even rarer, resulting in a lack of original research, except for very few specific entities [3,4,5,6]. Accordingly, only a small amount of mostly outdated research on the occurrence and predilection sites of childhood bone and soft-tissue tumors is available [7]. Despite extensive database research, using Pubmed, Cochrane Library and Google scholar, only one original research article about musculoskeletal tumors in children with precise data on the site of occurrence could be found. Published in 1990, Gebhard et al. presented data about tumors around the knee in children [8].

In bone tumors, the localization of lesions in the bone is very important in the diagnostic algorithm [9,10]. Specific entities are often located in specific predilection sites, which enables physicians to make a preliminary diagnosis using plain radiographs only [10,11]. However, this knowledge is still based on papers older than 40 years has not been confirmed in modern times [12,13,14]. Accordingly, most recent publications on childhood soft-tissue tumors focus on specific subgroups and their genetic or radiological profiles, while lacking general data on predilection sites [15,16,17,18,19].

The aim of this study was the retrospective assessment of predilection sites of benign and malignant childhood bone and soft-tissue tumors around the knee. This knowledge is critical to make a preliminary diagnosis and ultimately to accelerate the diagnostic work-up as well as the initiation of therapy.

## 2. Materials and Methods

This study was approved by the ethics committee of the Medical Faculty of the Technical University of Munich (N°48/20S; 21 March 2021). For the use of pseudonymized retrospective data, no written informed consent was needed.

For this retrospective study, the databank of our tertiary musculoskeletal cancer center was screened for children and adolescents treated for tumors of the leg between 2000 and 2021. To hold our data to the highest standards, only histopathologically verified tumors were included in this study. This resulted in the exclusion of all patients who had not undergone surgical treatment. Cases of watchful waiting in benign entities without biopsy, no-touch lesions or interventional treatments were not accounted for. The area around the knee was defined as the section extending from the distal third of the femur to the proximal third of the tibia.

X-rays were used to discern the locations of the bone tumors. The three patients suffering from multiple osteochondromas were excluded in these statistics. We differentiated between distal epi-, meta- and diaphyses of the femur and proximal epi-, meta- and diaphyses of the tibia and fibula, as well as the patella. To analyze the soft-tissue lesions, MR images were used. The MR protocols included high-resolution sequences in the short and long axes. The depth was classified into four categories: above the deep fascia, directly below the deep fascia, in the muscle tissue or in the knee joint.

From the database, sociodemographic data, the tumor location and the histopathological diagnosis were collected for each patient. The prevalence and predilection sites for all entities were calculated using SPSS software version 26 (SPSS, Inc., Chicago, IL, USA).

## 3. Results

Overall, 291 children between 0 and 18 years of age who underwent surgical treatment of primary bone and soft-tissue tumors around the knee were identified. A total of 67 patients were excluded due to insufficient data, leading to a total of 224 included subjects. One patient suffering from Ewing’s sarcoma was treated by definitive radiotherapy; all other patients with malignancies underwent surgical resection with (neo-)adjuvant chemo- or radiotherapy, if applicable. All included benign lesions underwent surgical treatment and received radical biopsies, marginal resection or curettage when suffering from pain. The sociodemographic data of the present cohort are summarized in Table 1. The mean age was 14.6 years (3 to 18 years) at the time of first surgical treatment, with a male-to-female ratio of 1.6:1. In total, 184 of 224 children presented with primary bone tumors, 40 with soft-tissue lesions (ratio of 4.6:1). More than 50% of all subjects were older than 15 years and only two patients were younger than 5 years at the time of diagnosis.

### 3.1. Bone Tumors

**Figure 1:**
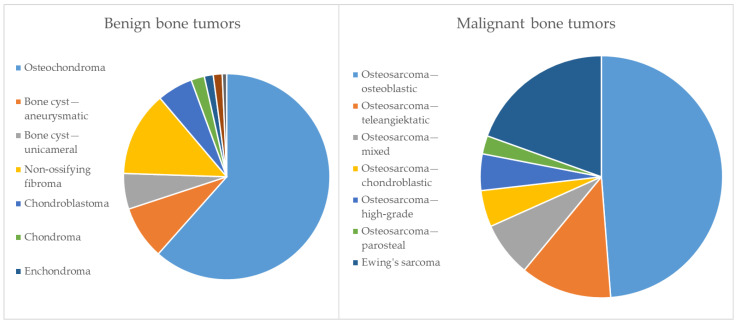
Entities and prevalence of bone tumors in study cohort.

The 184 children with bone tumors included 124 boys and 60 girls (2.1:1). Benign bone tumors were more than 3 times more common than malignant lesions (3.6:1). The entities and prevalence identified in the present cohort are summarized in Table 2 and visualized in Figure 1. The most common benign entity was osteochondroma (88). The rare benign entities were chondromas, enchondromas, giant-cell tumors of the bone and chondromyxoid fibroma. The predominant malignant entity was osteosarcoma (33), with conventional osteosarcomas being the most common sub-entity. Within the osteosarcomas, Grade 3 was diagnosed in 27 cases and Grade 2 in 6 cases. Most bone tumors were located in the femur (100), followed by the tibia (67), the fibula (12) and the patella (2). The tumor location within the long bones with respect to all identified entities is summarized in Table 3.

### 3.2. Soft-Tissue Tumors

**Figure 2:**
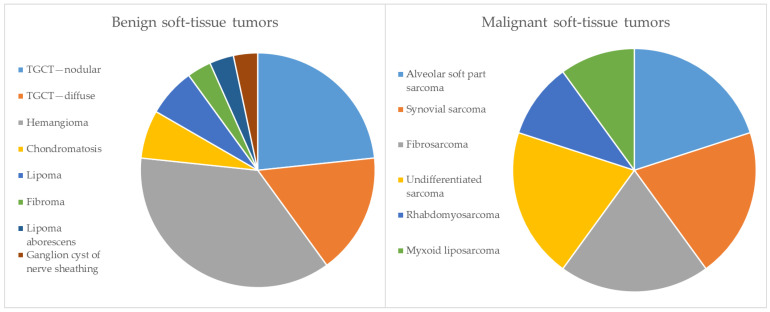
Entities and prevalence of soft-tissue tumors in study cohort.

Of the 40 soft-tissue tumors, 14 were found around boys’ and 26 around girls’ knees (0.7:1). The ratio of benign to malignant soft-tissue tumors was 3:1. Most children (12) with soft-tissue masses suffered from tenosynovial giant-cell tumors (TGCT), seven of whom presented with a localized type and five with a diffuse type (Table 4). The second most common entity was hemangioma in 11 cases. All other benign soft-tissue entities were significantly less common. Malignant soft-tissue tumors were extremely rare in the present cohort. The entities and prevalence of soft-tissue tumors are summarized in Table 4 and Figure 2. The grade was Grade 3 in three cases, Grade 2 in five cases and Grade 1 in two cases. Most soft-tissue tumors were located intra-articularly (17), and mainly TCGT, chondromatosis and lipoma arborescens were diagnosed. Additionally, one hemangioma and one synovial sarcoma were situated in the joint. A total of 11 tumors were found in muscles, mostly being hemangiomas (6), but four intramuscular sarcomas were also diagnosed. An overview of the localization pattern of childhood soft-tissue tumors is given in Table 5.

## 4. Discussion

The most important result of the present study is an updated map of predilection sites for tumors around the knee in children. Historical data on the sites of occurrence and prevalence could be confirmed for most entities.

The ratio of bone to soft-tissue tumors was almost identical compared to data published by Gebhardt et al. in 1990, despite a time span of over 30 years (4.6:1 vs. 5.2:1) [8]. Also confirming previously published data, an almost balanced gender distribution (1.6:1 vs. 1.2:1) could be identified in the present study [20,21]. According to previously published studies, bone and soft-tissue tumors are very rare under the age of ten, with most tumors occurring in the second decade of life [8,13,14,20,21]. As 93% of bone and 92% of soft-tissue tumors were diagnosed in patients over the age of ten in the present cohort, the previously published data could be completely confirmed. The lack of patients under the age of 5 in both this study and the study of Gebhardt et al. [8] can be explained by the design of this study. In particular, benign lesions (e.g., osteochondromas) are known to develop in small children, but are very seldom treated surgically and therefore not accounted for.

As expected, most bone tumors treated at our institution were osteochondromas, representing 47% of all tumors and 60% of benign bone lesions. All osteochondromas were located in the metaphyses of the long bones. Matching the results of Gebhardt et al. [8], non-ossifying fibromas were the second most common entity, representing 11% of all bone tumors. In contrast to their cohort, significantly more osteochondromas were diagnosed in the present cohort compared to NOF (ratio of 7.7:1 vs. 1.2:1). However, only patients who underwent surgical treatment were included in the present study. Due to the fact that most NOFs are incidental findings not requiring any therapy, the significantly lower proportion of NOFs can be explained. The prevalence of all rarer entities is comparable to the current literature [8,22]. Despite being a common primary benign bone tumor in children, as previously outlined by Gebhardt et al. [8], no osteoid osteomas were included in the present study. This can be explained by the typically interventional radiofrequency ablation of this entity at our institution, where no histopathological specimens are obtained. As was expected, no chondrosarcomas or bone metastases of other origins were found in our cohort. These two entities—being the two most common malignant masses in older patients’ bones—almost never occur in children [10,23].

In accordance with Phemister’s Law [24], most bone tumors were diagnosed in the long bones; lesions in the patella are very rare. Confirming the common principle that tumors typically occur at sites with high cellular activity, most bone lesions were found in the metaphysis. Regarding entity-specific predilection sites, the present results are in line with previously published diagrams [9]. In contrast to osteosarcoma, the predilection site for Ewing’s sarcoma is in the diaphysis. This aspect might lead to the exclusion of some cases due to the defined area of interest around the knee in the present study. Thus, a lower prevalence was identified compared to the published literature including all predilection sites. The prevalent site of metaphyses for osteosarcoma was confirmed in our cohort.

Confirming previous studies, only a small number of soft-tissue tumors could be identified in the present cohort [25]. Because of their status as tertiary center, the underrepresentation of small and superficial masses such as lipomas could be assumed, since patients with such tumors are often not referred to dedicated tumor centers.

Tenosynovial giant-cell tumors represented 40% of benign tumors and 30% of all soft-tissue masses, representing a much larger proportion compared to Gebhardt et al. (14%) [8]. On the other hand, Kransdorf et al. reported, in 1995, that TGCT is the most common soft-tissue tumor of the upper extremities [21]. Confirming previously published literature, all children suffering from TGCT were above the age of ten [19]. No desmoid tumors were found in our patients. Despite being the most frequent soft-tissue tumor in adults, lipomas are rarer in children and seldom treated surgically [23], which is confirmed by our data. All other benign soft-tissue entities showed a similar prevalence to that reported previously [8,21]. In accordance with Gebhardt and colleagues, only one rhabdomyosarcoma in 10 cases with malignant soft-tissue tumors was diagnosed, despite often being described as the most common malignant soft-tissue tumor in children [8,20]. Other malignant soft-tissue masses could be confirmed as rarities [18].

However, we have to note some limitations of this study. Firstly, most patients were referred to our dedicated tumor center. This could have led to an underrepresentation of small and clearly benign lesions. On the other hand, most published data are also from tertiary centers. Thus, this bias should be consistent throughout the sparse literature, so the results should be well comparable. Secondly, only histologically verified tumors were included in this study in order to ensure the correctness of the diagnoses, which led to an underrepresentation of benign lesions that can be diagnosed clinically and radiologically without previous biopsy. Thirdly, only 10 malignant soft-tissue tumors were diagnosed around the knee at our tertiary tumor center in a study period of over 20 years. Considering these small numbers, only few cases can significantly influence the results.

## 5. Conclusions

In the present study with a relatively large cohort considering the low incidence of these tumor entities, historically known predilection sites and prevalences for most bone and soft-tissue tumors were confirmed. In particular, the predominance of benign lesions and the occurrence of tumors in older children and adolescents was shown. The predilection site of the metaphyses was confirmed for most entities of bone tumors. Using this information, preliminary diagnoses can be made before surgical biopsy, possibly supporting a shorter diagnostic work-up and a faster initiation of therapy.

## Figures and Tables

**Table 1:** Sociodemographic data of study cohort.

		Male	Female	0–4 Years	5–9 Years	10–14 Years	15–18 Years	All
all		138	86	2	13	92	117	224
		62%	38%	1%	6%	41%	52%	
bone	all	124	60	1	11	77	95	184
tumors		67%	33%	1%	6%	42%	51%	
	benign	101	42	0	7	60	76	143
		71%	29%	0%	5%	42%	53%	
	malignant	23	18	1	4	17	19	41
		56%	44%	2%	10%	42%	46%	
soft tissue	all	14	26	1	2	15	22	40
tumors		35%	65%	3%	5%	38%	54%	
	benign	10	20	0	1	12	17	30
		33%	67%	0%	3%	40%	57%	
	malignant	4	6	1	1	3	5	10
		40%	60%	10%	10%	30%	50%	

**Table 2:** Entities and prevalence of bone tumors in study cohort.

Benign Bone Tumors			Malignant Bone Tumors		
Osteochondroma	88	62%	Osteosarcoma	33	80%
Bone cyst	20	14%	* - osteoblastic*	*20*	*49%*
* - aneurysmatic*	*12*	*8%*	* - teleangiektatic*	*5*	*12%*
* - unicameral*	*8*	*6%*	* - mixed*	*3*	*7%*
Non-ossifying fibroma	19	13%	* - chondroblastic*	*2*	*5%*
Chondroblastoma	8	6%	* - high-grade*	*2*	*5%*
Chondroma	3	2%	* - parosteal*	*1*	*2%*
Enchondroma	2	1%	Ewing‘s sarcoma	8	20%
Giant-cell tumors	2	1%			
Chondromyxoid fibroma	1	1%			
all	143		all	41	

**Table 3:** The predilection sites of bone tumors in the long bones around the knee. The three patients with multiple osteochondromas and the two aneurysmatic bone—cysts located in patellae are excluded from this statistic.

	Epiphyses		Metaphyses		Diaphyses	
	Patients	Percentage	Patients	Percentage	Patients	Percentage
Osteochondroma	0	0%	85	100%	0	0%
Bone cyst	0	0%	16	89%	2	11%
* * *- aneurysmatic*	*0*	*0%*	*9*	*90%*	*1*	*10%*
* * *- unicameral*	*0*	*0%*	*7*	*88%*	*1*	*12%*
Non-ossifying fibroma	0	0%	16	84%	3	16%
Chondroblastoma	0	0%	7	88%	1	12%
Chondroma	0	0%	3	100%	0	0%
Enchondroma	1	50%	1	50%	0	0%
Giant-cell tumors	1	50%	1	50%	0	0%
Chondromyxoid fibroma	0	0%	1	100%	0	0%
Osteosarcoma	3	9%	23	70%	7	21%
Ewing‘s sarcoma	0	0%	6	75%	2	25%
all	5	3%	159	89%	15	8%

**Table 4:** Entities and prevalence of soft-tissue tumors in the study cohort.

Benign Soft Tissue Tumors			Malignant Soft Tissue Tumors	
Tenosynovial giant-cell tumor	12	40%	Alveolar soft part sarcoma	2
* - nodular*	*7*	*23%*	Synovial sarcoma	2
* - diffuse*	*5*	*17%*	Fibrosarcoma	2
Hemangioma	11	37%	Undifferentiated sarcoma	2
Chondromatosis	2	7%	Rhabdomyosarcoma	1
Lipoma	2	7%	Myxoid liposarcoma	1
Fibroma	1	3%		
Lipoma aborescens	1	3%		
Ganglion cyst of nerve sheathing	1	3%		
all	30		all	10

**Table 5:** Localization pattern of benign soft-tissue tumors around the knee.

	Epifascial		Subfascial		Intramuscular		Intra-Articular	
	Patients	Percentage	Patients	Percentage	Patients	Percentage	Patients	Percentage
Tenosynovial giant-cell tumor	0	0%	0	0%	0	0%	12	100%
* * *- nodular*	*0*	*0%*	*0*	*0%*	*0*	*0%*	*7*	*100%*
* * *- diffuse*	*0*	*0%*	*0*	*0%*	*0*	*0%*	*5*	*100%*
Hemangioma	1	9%	3	27%	6	55%	1	9%
Chondromatosis	0	0%	0	0%	0	0%	2	100%
Lipoma	0	0%	1	100%	0	0%	0	0%
Fibroma	0	0%	1	100%	0	0%	0	0%
Lipoma aborescens	0	0%	0	0%	0	0%	1	100%
Ganglion cyst of nerve sheathing	0	0%	1	100%	0	0%	0	0%
Lymphangioma	0	0%	0	0%	1	100%	0	0%
all	1	3%	6	20%	7	23%	16	54%

## Data Availability

The raw data supporting the conclusions of this article will be made available by the authors on request.

## References

[1] Cunningham R.M., Walton M.A., Carter P.M. (2018). The Major Causes of Death in Children and Adolescents in the United States. N. Engl. J. Med..

[2] American Cancer Society (2014). Cancer Facts & Figures 2014. Special Section: Childhood & Adolescent Cancers.

[3] Paulino A.C., Nguyen T.X., Mai W.Y. (2007). An analysis of primary site control and late effects according to local control modality in non-metastatic Ewing sarcoma. Pediatr. Blood Cancer.

[4] Rastogi S., Prashanth I., Khan S.A., Trikha V., Mittal R. (2007). Giant cell tumor of bone: Is curettage the answer?. Indian J. Orthop..

[5] Balamuth N.J., Womer R.B. (2010). Ewing’s sarcoma. Lancet Oncol..

[6] Gassert F.G., Breden S., Neumann J., Gassert F.T., Bollwein C., Knebel C., Lenze U., von Eisenhart-Rothe R., Mogler C., Makowski M.R. (2022). Differentiating Enchondromas and Atypical Cartilaginous Tumors in Long Bones with Computed Tomography and Magnetic Resonance Imaging. Diagnostics.

[7] Little J. (1999). Epidemiology of Childhood Cancer.

[8] Gebhardt M.C., Ready J.E., Mankin H.J. (1990). Tumors about the knee in children. Clin. Orthop. Relat. Res..

[9] Miller T.T. (2008). Bone tumors and tumorlike conditions: Analysis with conventional radiography. Radiology.

[10] Freyschmidt J., Ostertag H. (2013). Knochentumoren: Klinik-Radiologie-Pathologie.

[11] Nichols R.E., Dixon L.B. (2011). Radiographic analysis of solitary bone lesions. Radiol. Clin. N. Am..

[12] Johnson L.C. (1953). A general theory of bone tumors. Bull. N. Y. Acad. Med..

[13] Schajowicz F. (1981). Tumors and Tumorlike Lesions of Bone and Joints.

[14] Dahlin D. (1978). Bone Tumors: General Aspects and Data on 6221 Cases.

[15] Mühlhofer H., Gersing A., Pfeiffer D., Wörtler K., Lenze U., Lenze F., Lallinger V., Haller B., Burgkart R., Von Eisenhart-Rothe R. (2020). Preoperative Evaluation of Myxofibrosarcoma: Prognostic Value and Reproducibility of Different Features on MRI. Anticancer Res..

[16] Logan S.J., Schieffer K.M., Conces M.R., Stonerock E., Miller A.R., Fitch J., LaHaye S., Voytovich K., McGrath S., Magrini V. (2021). Novel morphologic findings in PLAG1-rearranged soft tissue tumors. Genes Chromosomes Cancer.

[17] AMWF (2010). Guidlines Osteosarcoma. https://register.awmf.org/assets/guidelines/025-005l_S1_Osteosarkome_2021-11.pdf.

[18] Thacker M.M. (2013). Malignant soft tissue tumors in children. Orthop. Clin. N. Am..

[19] Thacker M.M. (2013). Benign soft tissue tumors in children. Orthop. Clin. N. Am..

[20] Kransdorf M.J. (1995). Malignant soft-tissue tumors in a large referral population: Distribution of diagnoses by age, sex, and location. AJR Am. J. Roentgenol..

[21] Kransdorf M.J. (1995). Benign soft-tissue tumors in a large referral population: Distribution of specific diagnoses by age, sex, and location. AJR Am. J. Roentgenol..

[22] Yildiz C., Erler K., Atesalp A.S., Basbozkurt M. (2003). Benign bone tumors in children. Curr. Opin. Pediatr..

[23] Hefti F. (2014). Kinderorthopädie in der Praxis.

[24] Phemister D.B. (1948). Cancer of the bone and joint. J. Am. Med. Assoc..

[25] Myhre-Jensen O. (1981). A consecutive 7-year series of 1331 benign soft tissue tumours: *Clinicopathologic data. Comparison with Sarcomas*. Acta Orthop. Scand..

